# Analytical and Clinical Performance of the CDC Real Time RT-PCR Assay for Detection and Typing of Dengue Virus

**DOI:** 10.1371/journal.pntd.0002311

**Published:** 2013-07-11

**Authors:** Gilberto A. Santiago, Edgardo Vergne, Yashira Quiles, Joan Cosme, Jesus Vazquez, Juan F. Medina, Freddy Medina, Candimar Colón, Harold Margolis, Jorge L. Muñoz-Jordán

**Affiliations:** Centers for Disease Control and Prevention, Division of Vector-Borne Diseases, Dengue Branch, San Juan, Puerto Rico, United States of America; University of California, Berkeley, United States of America

## Abstract

Dengue is an acute illness caused by the positive-strand RNA dengue virus (DENV). There are four genetically distinct DENVs (DENV-1–4) that cause disease in tropical and subtropical countries. Most patients are viremic when they present with symptoms; therefore, RT-PCR has been increasingly used in dengue diagnosis. The CDC DENV-1–4 RT-PCR Assay has been developed as an *in-vitro* diagnostic platform and was recently approved by the US Food and Drug Administration (FDA) for detection of dengue in patients with signs or symptoms of mild or severe dengue. The primers and probes of this test have been designed to detect currently circulating strains of DENV-1–4 from around the world at comparable sensitivity. In a retrospective study with 102 dengue cases confirmed by IgM anti-DENV seroconversion in the convalescent sample, the RT-PCR Assay detected DENV RNA in 98.04% of the paired acute samples. Using sequencing as a positive indicator, the RT-PCR Assay had a 97.92% positive agreement in 86 suspected dengue patients with a single acute serum sample. After extensive validations, the RT-PCR Assay performance was highly reproducible when evaluated across three independent testing sites, did not produce false positive results for etiologic agents of other febrile illnesses, and was not affected by pathological levels of potentially interfering biomolecules. These results indicate that the CDC DENV-1–4 RT-PCR Assay provides a reliable diagnostic platform capable for confirming dengue in suspected cases.

## Author Summary

Significant expansion of the four DENV serotypes (DENV-1, -2, -3 and -4) has been reported throughout tropical and sub-tropical regions of the world, with estimates of 390 million cases annually. The need has arisen for expanded diagnostic testing for DENV infections in the United States, as dengue infection has been added to the list of national notifiable diseases. Timely and accurate diagnosis of dengue is important for clinical care, disease surveillance, disease prevention, and control activities. However, current testing is performed with laboratory-developed research-based assays available only in a limited number of laboratories that have not been validated or approved for diagnostic testing in the United States. Here we report the development and evaluation of the CDC DENV-1–4 Real Time RT-PCR Assay, the first molecular test approved by the US Food and Drug Administration for the diagnosis and serotyping of DENV in human serum or plasma samples. This test was designed and validated for the detection of contemporary, clinically relevant DENV strains transmitted globally, facilitating the global deployment of the test and increase detection of traveler-associated dengue cases.

## Introduction

Dengue is a human disease caused by infection with any one of four genetically related dengue virus serotypes (DENV-1, -2, -3 and - 4), which are transmitted to humans by *Aedes* sp mosquitoes. Dengue is the most widespread mosquito-borne viral disease, with an estimated 390 million cases reported annually worldwide [1]–[3]. Infection by one DENV serotype confers long-term homotypic immunity but only transient heterotypic protection. Therefore, sequential heterotypic infections are common in dengue endemic areas [4]. In non-endemic regions of the United States (US), dengue is the most frequent cause of febrile illness among travelers returning from the Caribbean, Latin America, and Asia [5]–[8]. Occasional outbreaks occur in regions of the US where the mosquito vector is present, such as along the US-Mexico border and the states of Florida and Hawaii [9]–[14]. Frequent co-circulation of multiple DENV serotypes occurs in several US-associated territories where dengue is endemic, including Puerto Rico, the Virgin Islands, American Samoa, and other US-affiliated Pacific Islands [14]–[20].

Approximately 75% of DENV infections are acute, self-resolving, and asymptomatic [4]. Patients with dengue symptoms experience an illness that features three phases. The acute phase is characterized by fever lasting 2–7 days, which is often associated with one or more of the following symptoms: headache, retro-orbital eye pain, arthralgia, myalgia and/or bone pain, rash, mild hemorrhagic manifestations (e.g., nose or gum bleeding, petechiae, easy bruising) and leukopenia. The critical phase begins at defervescence, marking a 24–48 hour period in which plasma leakage, internal hemorrhage, severe organ impairment and compensated or decompensated shock may occur due to increased capillary permeability with plasma leakage, producing ascites, pleural effusions, and ‘third spacing’ of fluids [21]–[25]. These manifestations are currently termed severe dengue, a designation that has replaced dengue hemorrhagic fever (DHF) or dengue shock syndrome (DSS) [26]. Warning signs of severe dengue include abdominal pain, vomiting, thrombocytopenia, and mild to severe hemorrhagic manifestations. The convalescent phase of dengue may extend for several days and follows the febrile or critical phase of illness.

Early laboratory diagnosis of dengue is best made during the acute phase of the illness when viremia levels are high (0–5 days post onset of illness) and the viral RNA or soluble antigens (i.e. NS1 antigen) can be detected [24], [27]–[35]. IgM anti-DENV only becomes detectable 4 or 5 days post symptoms onset. Several RT-PCR assays, including the increasingly common real-time systems, have been developed for DENV detection and serotype identification with a high level of sensitivity and specificity [32], [34], [36]–[38]. Coupled with automated RNA extraction protocols, these assays can now facilitate the faster and more accurate diagnosis of dengue suspected cases. It has been a common practice that an RT-PCR negative result during the acute phase of illness requires the collection of a second, convalescent sample to be tested for the presence of IgM anti-DENV antibodies. However, with RT-PCR assays becoming more sensitive, faster and affordable, it is now more feasible to diagnose dengue on the single sample obtained during the patient's first visit to the clinician.

Among each DENV serotype, several distinct genetic lineages designated as genotypes have been identified and differ by approximately 5–6% in their genomic sequence [39]–[41]. This genotype variability has been associated with the evolution of DENV over a wide geographic distribution and occasionally with epidemic potential [42]–[44]. Consequently, molecular diagnostic tools should consider the genomic sequence variability among circulating strains of current genotypes.

Nucleic acid detection tests for dengue diagnosis were first developed over 20 years ago [45] and have been generally used for research, but not validated for diagnostic use in the US or globally. Although the most effective method to diagnose dengue in the acute phase of the illness recommended by the WHO (WHO dengue guidelines 2009) is detection of DENV RNA, widespread use of dengue molecular diagnostics has been hampered by lack of validated tests and testing capability, perceptions that molecular diagnostics are cost prohibitive compared to immunoassays and lack of recommendations for their use.

To meet some of the challenges that have limited the use of dengue molecular diagnostics in the US and the world, we developed the CDC DENV-1–4 Real-Time RT-PCR Assay and carried it thorough the FDA 510K process for approval as an *in vitro* diagnostic device for human patient diagnosis of dengue. This test runs on the same instrumentation, software, and reagents used by other Centers for Disease Control and Prevention (CDC) diagnostic platforms approved by the FDA, such as the CDC Human Influenza Real-Time RT-PCR Diagnostic Panel (cat# KT0096) (http://www.cdc.gov/media/releases/2011/p0902_diag_lab.html). Here, we report the performance of this assay, including its capability to detect DENV from multiple lineages, and the clinical sensitivity and specificity for diagnosis in dengue suspected human cases.

## Materials and Methods

### Ethics Statement

Serum samples for the clinical performance studies were obtained from the Dengue Surveillance System sample collection under guidelines approved by the CDC institutional review board (IRB). Patients did not provide verbal or written consent for this study. Samples were de-linked from patient identifiers according to CDC's IRB.

### Dengue Virus Isolates

Several DENV panels were used to evaluate the analytical performance of the RT-PCR Assay. A 5 replica panels of eight 1∶10 dilutions of quantified (GCE/mL) laboratory-adapted, heat inactivated DENV strains (DENV-1 Haw, DENV-2 NGC, DENV-3 H87, DENV-4 H241) were prepared in human serum or plasma (Bioreclamation Inc). A second panel consisted of 29 DENV-1–4 isolates obtained from serum samples from patients residing in different countries world-wide, cultured, and quantified as described previously [46]. Briefly, serum was inoculated into C6/36 (*Aedes albopictus*) cell culture maintained in Dulbecco's Minimal Essential Medium and incubated at 33°C for 5 days. The quantified stocks were diluted in human serum at 1∶10 dilutions down to 10^2^ GCE/mL. Triplicate samples of each dilution were tested. Serum samples were collected under guidelines approved by the CDC IRB.

### Virus Stock Quantification

*In vitro*-transcribed RNA was used as the copy number control to quantify stocks of laboratory-adapted DENV strains as previously described [47]. Briefly, templates were developed by amplifying DENV-1–4 using the same corresponding primer pairs in the CDC DENV-1–4 Real Time RT-PCR Assay (Table 1) and cloned into a TOPO TA vector (Invitrogen, cat# K4600-01). Target RNA was transcribed with T7 RNA polymerase using AmpliScribe T7 Flash Transcription Kit (Epicentre, cat# ASF3257). The resulting RNA was quantified by spectrophotometry and expressed as genome copy equivalents per mL (GCE/mL).

**Table 1:** Comparison of published and oligonucleotides used in CDC DENV-1–4 Real Time RT-PCR Assay.

	Oligonucleotide		Frequency*	Position	Gene	Length	Amplicon size
D1-F	Published (1)	CAAAAGGAAGTCGTGCAATA	39/48	8936–8955	NS5	20	112 bp
	CDC DENV-1–4 Real Time RT-PCR	CAAAAGGAAGTCG**Y**GCAATA	48/48				
D1-R	Published (1)	CTGAGTGAATTCTCTCTACTGAAC	10/48	9023–9047	NS5	25	112 bp
	CDC DENV-1–4 Real Time RT-PCR	CTGAGTGAATTCTCTCT**G**CT**R**AAC	48/48				
D1-Probe	Published (1)	CATGTGGTTGGGAGCACGC	16/48	8961–8979	NS5	19	112 bp
	CDC DENV-1–4 Real Time RT-PCR	CATGTGG**Y**TGGGAGC**R**CGC	48/48				
D2-F	Published (1)	CAGGTTATGGCACTGTCACGAT	0/50	1426–1447	E	22	78 bp
	CDC DENV-1–4 Real Time RT-PCR	CAGG**C**TATGGCAC**Y**GTCACGAT	40/50				
D2-R	Published (1)	CCATCTGCAGCAACACCATCTC	6/50	1482–1504	E	22	78 bp
	CDC DENV-1–4 Real Time RT-PCR	CCAT**Y**TGCAGCA**R**CACCATCTC	48/50				
D2-Probe	Published (1)	CTCTCCGAGAACAGGCCTCGACTTCAA	3/50	1454–1480	E	27	78 bp
	CDC DENV-1–4 Real Time RT-PCR	CTC**Y**CC**R**AGAAC**G**GGCCTCGACTTCAA	43/50				
D3-F	Published (1)	GGACTGGACACACGCACTCA	9/51	701–720	prM	20	74 bp
	CDC DENV-1–4 Real Time RT-PCR	GGACT**R**GACACACGCAC**C**CA	50/51				
D3-R	Published (1)	CATGTCTCTACCTTCTCGACTTGTCT	21/51	749–775	prM	26	74 bp
	CDC DENV-1–4 Real Time RT-PCR	CATGTCTCTACCTTCTCGACTTG**Y**CT	51/51				
D3-Probe	Published (1)	ACCTGGATGTCGGCTGAAGGAGCTTG	50/51	722–747	prM	20	74 bp
	CDC DENV-1–4 Real Time RT-PCR	ACCTGGATGTCGGCTGAAGGAGC**T**TG	50/51				
D4-F	Published (1)	TTGTCCTAATGATGCTGGTCG	12/18	884–904	prM	21	89 bp
	CDC DENV-1–4 Real Time RT-PCR	TTGTCCTAATGATGCT**R**GTCG	17/18				
D4-R	Published (1)	TCCACCTGAGACTCCTTCCA	13/18	953–973	prM	20	89 bp
	CDC DENV-1–4 Real Time RT-PCR	TCCACC**Y**GAGACTCCTTCCA	18/18				
D4-Probe	Published (1)	TTCCTACTCCTACGCATCGCATTCCG	12/18	939–965	prM	26	89 bp
	CDC DENV-1–4 Real Time RT-PCR	T**Y**CCTAC**Y**CCTACGCATCGCATTCCG	18/18				

Note: The sequences of the primers and probes of CDC DENV-1-4 RT-PCR Assay for detection of DENV-1-4 are covered under a pending patent application.

* Frequency: number of complete sequence perfect matches over the total number of sequences screened.

### CDC DENV-1–4 Real Time RT-PCR Assay

The Assay includes a set of oligonucleotide primers and dual-labeled 5′-fluorescent (Taqman) probes for *in vitro*, qualitative detection of DENV-1–4. The targeted regions of DENV RNA are transcribed into complimentary DNA (cDNA) and amplified by the polymerase chain reaction (PCR). The fluorophore-labeled probes anneal to amplified DNA fragments and the fluorescent signal intensity is monitored by the ABI 7500 Fast Dx instrument during each PCR cycle. Target amplification is recorded as increase and accumulation of fluorescence over time in contrast to background signal. The assay can be run in singleplex (each DENV serotype detected in a separate reaction) or in multiplex (the four DENV serotypes are run in the same reaction) formats facilitated by the targeting of each DENV serotype with a different colored probe, i.e. each Taqman probe targets a single DENV serotype and is conjugated to a fluorophore that emits fluorescence at different excitation wavelengths: 5′-FAM DENV-1 BHQ1-3′, 5′HEX DENV-2 BHQ1-3′, 5′-TR DENV-3 BHQ2-3′, and 5′-Cy5 DENV-4 BHQ3-3′. All validations in this study were performed using the CDC DENV-1–4 Real Time RT-PCR Assay in accordance to the manufacturer's instructions (Package Insert, KK0128 available at www.cdc.gov/dengue). Viral RNA was extracted using the QIAamp DSP Viral RNA Mini kit (Qiagen cat# 61904) following manufacturer's suggested protocol. All RT-PCR reactions were performed and validated using SuperScript III Platinum One-Step Quantitative RT-PCR System without Rox (Invitrogen, cat# 11732-088). To assemble a multiplex RT-PCR reaction, 5 µL of RNA were mixed with the following reagents: 2.2 µL of nuclease-free H_2_O, 12.5 µL of 2× premix, 0.5 µL of forward and reverse primers for DENV-1 and DENV-3 (final concentration 1 µM), 0.25 µL of forward and reverse primers for DENV-2 and DENV-4 (final concentration 500 nM), 0.45 µL of each Taqman probe (final concentration 180 nM), and 0.5 µL of SuperScript III RT/Platinum Taq mix to a final reaction volume of 25 µL. Individual reactions were run in either 8-tube optical strips or 96-well plates and placed in the ABI 7500 FAST Dx thermocycler (Applied Biosystems, cat# 4406985). The standard cycling method was selected and fluorescence capture set to detect emissions through the FAM, HEX, Texas Red, and CY5 channels in each well. Thermocycling parameters were as follows: reverse transcription (RT) at 50°C for 30 min, RT inactivation at 95°C for 2 min and fluorescence detection for 45 cycles of 95°C for 15 seconds and annealing at 60°C for 1 min. Amplification curves were evaluated by serotype and the threshold line placed above overt background signal, usually intersecting the initial exponential phase of the curve for each serotype individually. Amplification curves with CT values >37 render erratically and are difficult to ascertain with increasing CT values; therefore present unreliable results and were considered negative.

### RNA Extraction Methods

Several viral RNA extraction methods, manual or automated, were validated for use with this assay. The QIAamp DSP Viral RNA Mini kit (Qiagen, cat# 9001292), used manually or in the automated QIAcube and the MagNA Pure LC 2.0 automated system (Roche, cat# 05-197-686-001) were extensively validated. Additional kits and platforms were tested and found to have similar performance, including QIAamp Viral RNA mini kit (Qiagen, cat#s 52904 and 52906) manual or on QIAcube; the automated BioRobot Universal System (Qiagen, cat# 9001094); the automated BioRobot M48 System (Qiagen, cat# 9000708).

### Sequence and Phylogenetic Analysis

Sequencing of the DENV envelope glycoprotein (E) gene was performed as a comparative method to confirm diagnostic results obtained with the assay. The E gene (1,485 bp) from every isolate was sequenced. Briefly, E gene was amplified using BluePrint One-Step RT-PCR kit (Takara #RR755A) with serotype-specific primers following manufacturer suggested protocol. The sequences were determined by BigDye Terminator v3.1 Cycle sequencing kit (Life Technologies, cat#4337455) following manufacturer suggested protocol and bi-directional Sanger method. All amplification and sequencing primers are listed in Supporting Table S1. The coding sequences were assembled using SeqMan found in DNASTAR Lasergene 9 Core Suite (http://www.dnastar.com/t-products-lasergene.aspx). All sequence alignments were performed using the ClustalW module from MEGA5 (www.megasoftware.net). Phylogenetic analyses were performed by generating maximum likelihood trees under the GTR+I+Γ4 nucleotide substitution model available in MEGA5. GenBank accession numbers are available upon request.

### Clinical Performance Studies

A total of 86 acute serum samples were collected from febrile patients in Puerto Rico and Costa Rica (2009–2011) suspected of having dengue 0–5 days after onset of symptoms and whose average age was 14.3 years, in a study approved by the CDC IRB. Viral RNA was extracted using the Qiagen QIAamp DSP Viral RNA Mini Kit following the manufacturer's protocol. Viral RNA was tested by the multiplex format of the RT-PCR Assay and results were confirmed by DENV E gene sequencing. No convalescent specimens were obtained from these patients to test for IgM anti-DENV seroconversion. The CDC DENV-1–4 Real-Time RT-PCR Assay was further evaluated by testing 371 archived, paired acute and convalescent serum samples obtained (2007–2011) from CDC's routine passive dengue surveillance system in Puerto Rico and 19 countries: Trinidad and Tobago, St. Martin, Venezuela, Mexico, Colombia, Brazil, Dominican Republic, India, Barbados, Samoa, Haiti, Ecuador, Costa Rica, Grenada, Honduras, Thailand, and other Netherland Antilles in accordance with a CDC IRB approved protocol. The convalescent samples, all collected at least 6 days after onset of symptoms, were tested with the IgM anti-DENV Capture Enzyme Linked Immunosorbent Assay (CDC MAC-ELISA) [48].

### Specificity Studies

The following twelve organisms in the differential diagnosis of patients with a febrile illness suspected of dengue were selected for the specificity validation: West Nile Virus (WNV), yellow fever virus (YFV), St. Louis encephalitis virus (SLEV), Chikungunya virus (CHIKV), herpes simplex virus 1 and 2 (HSV-1-2), cytomegalovirus (CMV), varicella zoster virus (VZV), and bacterial organisms *Leptospira*, and *Borrelia*. These pathogens were obtained from CDC repositories.

### Endogenous Interference

Laboratory-grade, human endogenous interferent biomolecules were obtained and diluted in normal human serum to the following physiologically relevant pathological concentrations: bilirubin (342 µmol/L), cholesterol (13 mmol/L), hemoglobin (2 g/L), triglycerides (37 mmol/L) and genomic DNA (400 µg/100 mL). These concentrations follow guidelines published by the National Committee for Clinical Laboratory Standards (NCCLS), EP7-A2 (2005).

## Results

### Assay Design

The CDC DENV-1–4 Real-Time RT-PCR Assay was designed to detect a wide variety of current clinically relevant strains of DENV-1, -2, -3, and -4 RNA in serum or plasma. Each probe was labeled with a distinct fluorophore-color allowing the simultaneous detection of the four DENV serotypes in a single reaction. The RT-PCR reactions can be assembled in singleplex or multiplex formats; both of these formats have been shown to provide equivalent sensitivity. The assay kit includes a positive control virus mix, which consists of heat-inactivated DENV-1 Haw, DENV-2 NGC, DENV-3 H87, and DENV-4 H241, and an assay internal control, termed Human Specimen Control (HSC). HSC consists of non-infectious cultured human cell material derived from the A549 cell line supernatant that tests positive for the presence of human RNaseP RNA (RP). RP RNA is present in most clinical samples and a positive signal confirms the integrity of the extraction reagents and the successful recovery of RNA. The sequence of primers and probes for both, HSC and RP, were obtained from the CDC Human Influenza Virus Real Time RT-PCR Diagnostic Panel (cat# KT0096).

**Figure 1:**
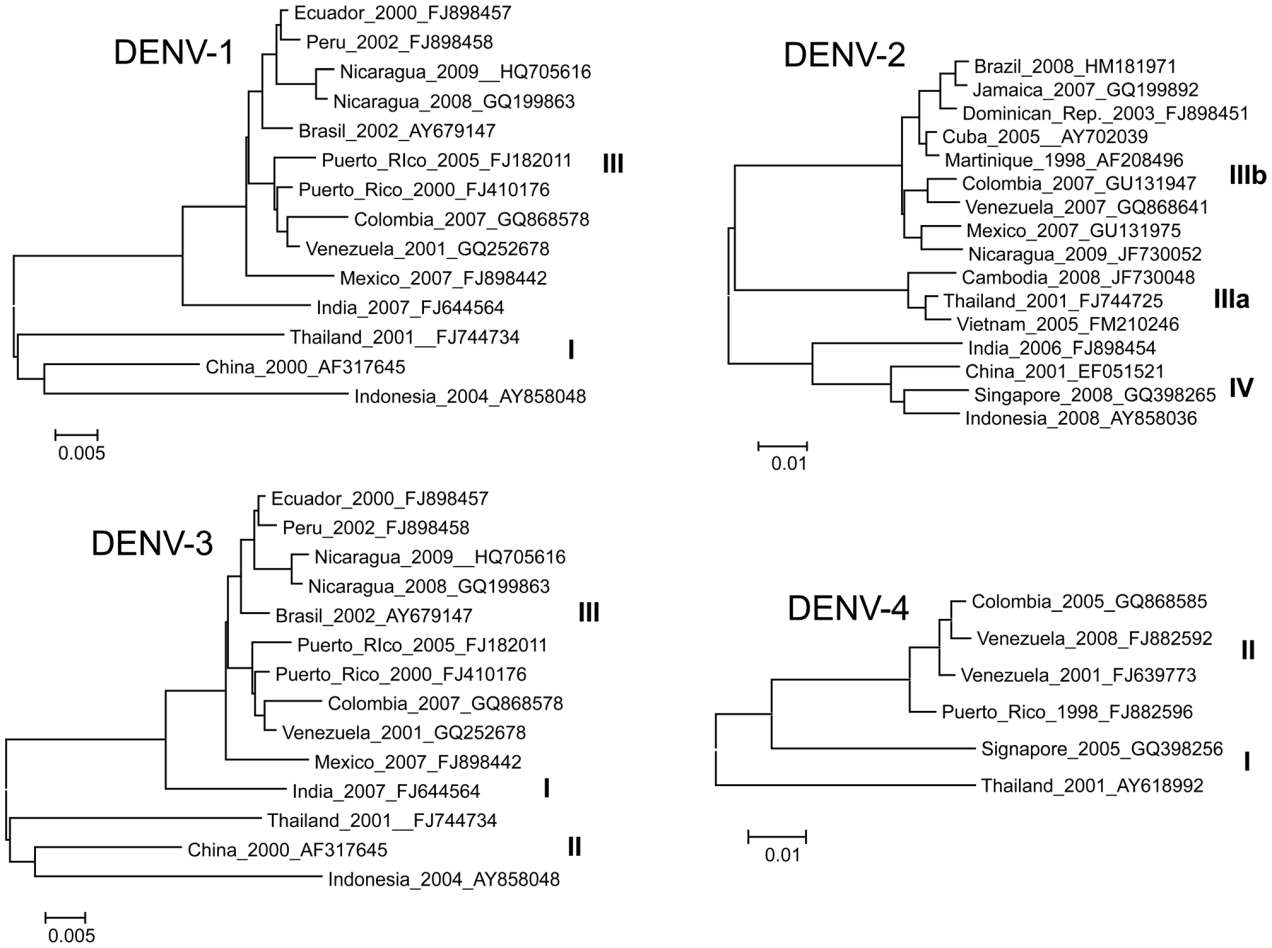
Maximum likelihood phylogeny of contemporary DENV strains. Representative DENV strains from currently circulating genotypes considered for primer and probe modifications. Full genome sequences were used. Each taxa is labeled: country of origin, year of isolation, and GenBank accession number. Due to figure space limitations, only a representative subset of strains were used to generate these ML trees. DENV-1 Asian (I) and American-African (III) genotypes [55], DENV-2 Asian I (IIIa), American-Asian (IIIb), and Cosmopolitan (IV) genotypes [42], DENV-3 South Pacific (I), Thailand (II), and Indian Subcontinent (III) genotypes [55], [56], DENV-4 Southeast (I) and Indonesian (II) genotypes [56].

The primers and probes used in the Assay were originally based on sequences previously published by Johnson et al., 2005 [34], however, significant modifications were introduced to increase complementarity with contemporary DENV sequences available in GenBank (Table 1). We selected 48 DENV-1, 50 DENV-2, 51 DENV-3, and 18 DENV-4 complete genome sequences as design templates from viruses isolated between 1998–2012, representing contemporary genotypes, and covering a wide geographical distribution. Figure 1 shows maximum likelihood trees representing the phylogeny of currently circulating lineages of each DENV serotype. Every primer and probe of the new CDC DENV-1–4 RT-PCR was compared to the corresponding serotype alignment and determined the frequency of every base in the oligonucleotide. Mismatches between PCR oligonucleotides and the sequence alignments were attended to as follows: degeneracy was introduced in a given nucleotide position when mismatch was 30% or greater between 2 or more strains; a base was replaced by another fixed base when mismatch was 90% or greater between 2 or more strains in a given position. International Union of Pure and Applied Chemistry (IUPAC) codes were used for all degenerate bases. The modified primer and probe sequences in the CDC DENV-1–4 Real-Time RT-PCR Assay are shown in Table 1 compared to the previously published sequences. The frequency in which each nucleotide position is matched to the representative strains of every serotype is also indicated. Additional information includes: target gene per serotype, target positions of every oligonucleotide relative to genome, and length of the amplicons. We found low probability of homodimer or heterodimer formation using OligoAnalyzer 3.1 (www.idtdna.com/analyzer) with predicted ΔG≥−9 kcal/mol between most oligonucleotide pair combination considering multiplex reaction conditions. This was confirmed experimentally running multiple oligonucleotide combinations in non-template control reactions and after the evaluation of all our clinical and analytical sample panels.

### Performance Comparison

**Figure 2:**
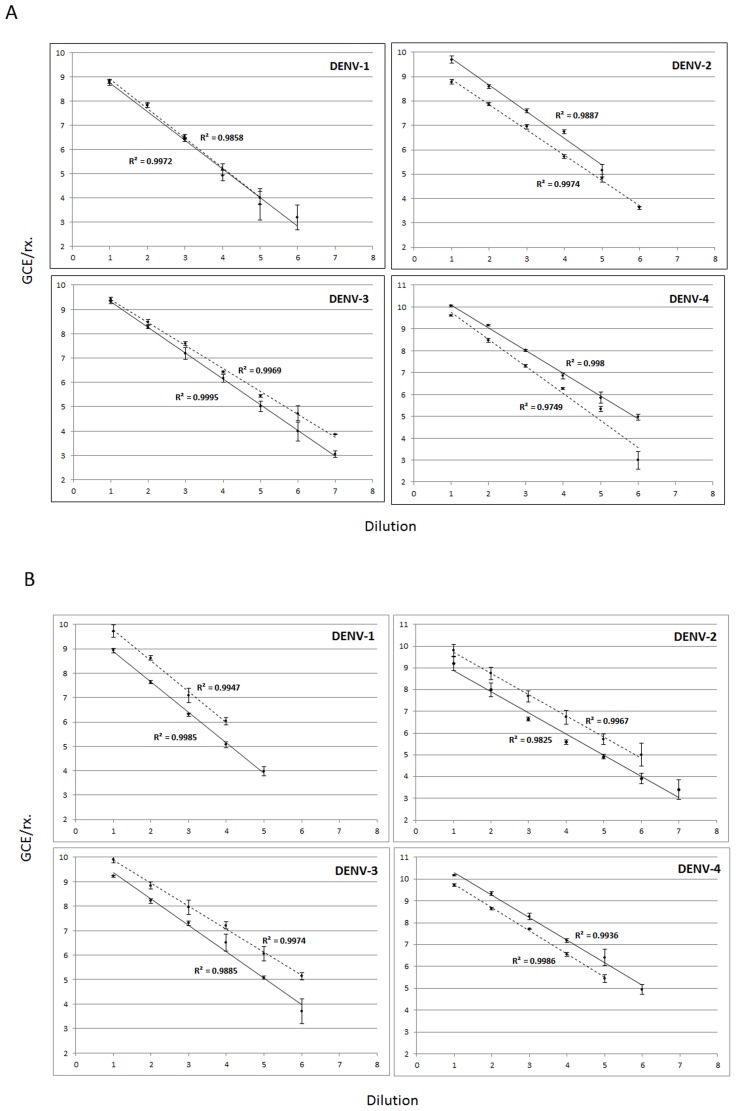
Performance comparison. Performance and sensitivity of the assay was compared to the Johnson et al., 2005 real time RT-PCR assay by detection of (8) ten-fold dilutions of *in vitro* transcribed RNA derived from target regions, 5 replicas per dilution. Both assays, CDC and Johnson 2005, were performed in singleplex **(A)** and multiplex **(B)** formats. Viral RNA was quantified using a standard curve measured in genome copy equivalents per RT-PCR reaction (GCE/rx) log transformed for linear scale plotting. Each data point reflects the average CT of 5 replicates. A linear regression was estimated for CDC (black solid lines) and Johnson 2005 (black dashed lines). Regression coefficient (R^2^) is shown for every regression and every serotype. Error bars indicate standard deviation.

Once the Assay was designed and optimized, we compared performance and sensitivity against the real time RT-PCR assay designed by Johnson et al., 2005. *In vitro* transcribed RNA derived from the target regions was used as template and spiked normal human serum: 8 serial dilutions 1∶10, 5 replicates. Both assays were simultaneously run with the same samples in singleplex and multiplex formats according to each suggested protocol. The concentrations of virus RNA detected were measured in GCE/reaction. The CDC Real Time DENV-1–4 RT-PCR assay outperformed the Johnson 2005 assay detecting 10-fold less target RNA in 100% of the replicates in DENV-1 and DENV-2 singleplex format and DENV-1, -2, and -4 in multiplex format (Figure 2A and B respectively). Efficiency of amplification and overall performance was similar between both assays.

### Limit of Detection

**Figure 3:**
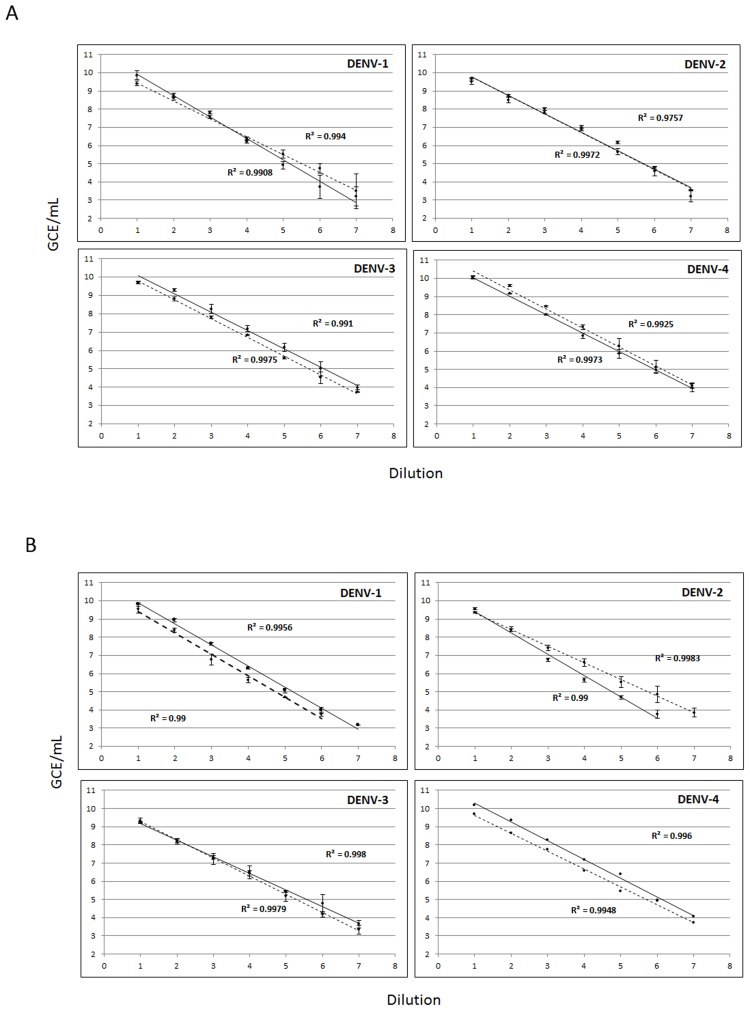
Limit of detection. The limit of detection of the Assay was evaluated by detection and quantification of (8) ten-fold dilutions of laboratory-adapted DENV-1–4 strains, 5 replicas per dilution. The Assay was performed in singleplex **(A)** and multiplex formats **(B)**. Viral RNA concentration was quantified using a standard curve measured in genome copy equivalents per milliliter of serum or plasma (GCE/mL). A linear regression was estimated for dilutions of virus stocks in plasma (dashed lines) or serum (solid line). Regression coefficient (R^2^) is shown for each serotype. Error bars indicate standard deviation of measurements from 5 replicas in GCE/mL.

The limit of detection (LoD) was defined as the last dilution in which virus was detected in all replicates and 100% reproducibility can be guaranteed. The LoD of the assay was determined using a panel of quantified (GCE/mL) DENV stocks diluted in human serum or plasma for eight 1∶10 dilutions with 5 replicates of each dilution. Viral RNA from every replicate was extracted and tested following the package insert protocol. The RT-PCR Assay was run quantitatively to compare the LoD of different assay formats. The amount of serum sample processed for RNA extraction, the RNA elution volume, and the amount of RNA elution added per RT-PCR reaction were considered in order to calculate the viral RNA concentration per reaction and converted to units per mL of serum. Virus detection, measured in genome copy equivalents per mL of sample (GCE/mL), was compared between virus dilutions in human serum and plasma in singleplex and multiplex formats (Figures 3A and B, respectively). Viral RNA was occasionally detected in dilutions with 1×10^2^-1×10^1^ GCE/mL; however the LoD was determined to be between 1×10^4^ and 1×10^3^ GCE/mL for all serotypes in both formats in serum and plasma.

Viral RNA extractions were performed using the Qiagen QIAamp DSP Viral RNA Mini Kit in the initial evaluations and compared with other manual or automated viral RNA extraction kits. The performance of each kit was measured by extracting viral RNA from a similar panel of viruses diluted in human serum. The lowest titer of RNA detection from each kit was determined as shown in Supporting Table S2. Virus concentrations at the LoD dilution (1×10^3^ GCE/mL) or at a 1∶10 dilution below the LoD (1×10^2^ GCE/mL) were detected for each virus-type. These results validate comparable levels of DENV-1–4 RNA detection after viral RNA extraction with the selected methods.

The RT-PCR Assay was evaluated to determine detection of contemporary DENV strains from a broad geographical distribution at a LoD comparable to the reference panel described above. 29 DENV-1–4 strains from global locations were cultured, quantified and serial 1∶10 dilutions in human serum down to 1×10^2^ GCE/mL were tested in triplicate. The observed LoD was similar to that obtained from the reference panel described above (Table 2).

**Table 2:** Detection of international strains: (10^4^ GCE/mL) above and below (10^2^ GCE/mL) the limit of detection.

				10^4^ GCE/ml	10^2^ GCE/ml
Serotype	YEAR	Country	Genotype	Rate Pos	Rate Pos
DENV-1	2003	Brazil	African/American	3/3	0/3
DENV-1	2007	Mexico	African/American	3/3	0/3
DENV-1	2007	Venezuela	African/American	3/3	0/3
DENV-1	1994	Sri Lanka	South Pacific	3/3	0/3
DENV-1	2004	Philippines	South Pacific	3/3	0/3
DENV-1	2004	Thailand	Asian	3/3	0/3
DENV-1	2006	Thailand	Asian	3/3	0/3
DENV-2	2003	Brazil	SE Asian/American	3/3	0/3
DENV-2	2007	Colombia	SE Asian/American	3/3	0/3
DENV-2	1980	Ivory Cost	Sylvatic	3/3	2/3
DENV-2	1988	Vietnam	Asian II	3/3	1/3
DENV-2	2006	Thailand	Asian II	3/3	0/3
DENV-2	2003	Dominican R.	SE Asian/American	3/3	1/3
DENV-2	2003	Costa Rica	SE Asian/American	3/3	0/3
DENV-2	1996	Peru	American	3/3	1/3
DENV-2	1982	Burkina Faso	Cosmopolitan	3/3	1/3
DENV-2	2006	India	Cosmopolitan	3/3	0/3
DENV-3	2006	Puerto Rico	Indian Subcont.	3/3	0/3
DENV-3	2003	Brazil	Indian Subcont.	3/3	1/3
DENV-3	1995	Samoa	South Pacific	3/3	0/3
DENV-3	2006	Thailand	Thailand	3/3	0/3
DENV-3	2000	Ecuador	Indian Subcont.	3/3	1/3
DENV-3	1991	Cook Island	South Pacific	3/3	0/3
DENV-4	2006	Colombia	Indonesian	3/3	0/3
DENV-4	2006	Mexico	Indonesian	3/3	0/3
DENV-4	1992	Sri Lanka	SE Asian	3/3	1/3
DENV-4	2006	Thailand	SE Asian	3/3	0/3
DENV-4	1994	St. Croix	Indonesian	3/3	0/3
DENV-4	1999	Ecuador	Indonesian	3/3	1/3
DENV-4	1995	Micronesia	SE Asian	3/3	0/3

### Cross Reactivity

The analytical specificity of the assay was evaluated by testing the nucleic acid extracted from the 12 organisms described in Methods, in triplicate, to determine if a positive signal was generated compared to samples spiked with DENV. No sample, except those containing DENV, produced a positive signal; therefore no cross-reaction was observed (Table 3).

**Table 3:** CDC DENV-1–4 Real Time RT-PCR Assay specificity against clinically relevant concentrations of other pathogens.

Pathogen	Sample type	Concentration	DENV RT-PCR Rate positive
**Virus**		**pfu/mL**	
DENV-1	spiked serum	1×10^6^	3/3
DENV-2	spiked serum	1×10^6^	3/3
DENV-3	spiked serum	1×10^6^	3/3
DENV-4	spiked serum	1×10^6^	3/3
West Nile Virus	spiked serum	6.9×10^7^	0/3
Yellow Fever Virus	spiked serum	3.7×10^6^	0/3
St. Louis Encephalitis Virus	spiked serum	3.7×10^6^	0/3
Chikungunya Virus	spiked serum	4.0×10^6^	0/3
Hepatitis C Virus	clinical serum specimen	unknown	0/3
Hepatitis A Virus	clinical serum specimen	unknown	0/3
Herpes Simplex 1 Virus	spiked serum	1.0×10^5^	0/3
Herpes Simplex 2 Virus	spiked serum	1.0×10^5^	0/3
Cytomegalovirus	spiked serum	1.0×10^5^	0/3
Varicella Zoster Virus	spiked serum	1.0×10^5^	0/3
**Bacteria**		**bacteria/mL**	
*Leptospira*	spiked serum	2.5×10^5^	0/3
*Borrelia burgdorferi*	spiked serum	1.0×10^6^	0/3

### Interference

In order to evaluate if DENV RNA detection is interfered by the presence human endogenous biomolecules, pathological concentrations of a selection of biomolecules were prepared in normal human serum. Each preparation was then spiked with the cultured DENV-1–4 laboratory-adapted strains mentioned above at concentrations of 1∶10 above the LoD (1×10^4^ GCE/mL), equal to the LoD (1×10^3^ GCE/mL), and 1∶10 below the LoD (1×10^2^ GCE/mL). None of the tested biomolecules showed any significant interference in DENV detection (Table 4).

**Table 4:** Detection of DENV in the presence of potentially interfering substances present in blood.

		Normal Human Serum			Bilirubin (342 µmol/L)			Cholesterol (13 mmol/L)		
		AVG CT	STD	Rate Pos	AVG CT	STD	Rate Pos	AVG CT	STD	Rate Pos
DENV-1	Above LoD	33.47	1.97	3/3	29.48	0.64	3/3	29.23	1.23	3/3
	LoD	35.33	0.88	3/3	32.37	0.20	3/3	34.89	1.09	3/3
	Below LoD	37.93	0.00	0/3	35.86	2.11	2/3	ND	ND	0/3
DENV-2	Above LoD	33.59	0.69	3/3	32.39	0.78	3/3	34.10	0.10	3/3
	LoD	35.44	1.90	3/3	35.79	2.71	3/3	36.62	3.83	2/3*
	Below LoD	35.87	0.00	1/3	35.84	0.50	3/3	36.95	1.45	1/3
DENV-3	Above LoD	32.00	0.76	3/3	31.32	0.21	3/3	33.03	0.72	3/3
	LoD	34.33	1.73	3/3	35.37	2.40	3/3	34.05	0.96	3/3
	Below LoD	33.18	0.00	1/3	34.98	3.21	2/3	36.34	1.92	1/3
DENV-4	Above LoD	31.99	0.58	3/3	29.59	0.43	3/3	29.64	1.01	3/3
	LoD	35.17	0.24	3/3	32.27	0.30	3/3	33.93	0.13	3/3
	Below LoD	36.37	0.00	1/3	36.19	1.08	3/3	42.30	0.00	0/3

		Hemoglobin (2 g/L)			Triglycerides (37 mmol/L)			Genomic DNA (400 µg/100 mL)		
		AVG CT	STD	Rate Pos	AVG CT	STD	Rate Pos	AVG CT	STD	Rate Pos
DENV-1	Above LoD	29.13	0.21	3/3	31.66	1.01	3/3	32.63	1.20	3/3
	LoD	32.56	0.80	3/3	35.46	0.86	2/3	34.75	0.58	3/3
	Below LoD	38.17	0.00	0/3	38.15	0.00	0/3	39.02	0.00	0/3
DENV-2	Above LoD	32.65	0.17	3/3	33.04	1.66	3/3	32.83	1.07	3/3
	LoD	35.80	0.71	2/3	35.46	1.41	3/3	35.95	0.80	3/3
	Below LoD	37.44	2.96	3/3	39.11	0.00	0/3	36.62	0.00	1/3
DENV-3	Above LoD	32.08	0.68	3/3	34.60	1.15	3/3	36.99	4.49	3/3
	LoD	34.30	0.47	3/3	37.12	0.00	0/3	39.14	0.00	0/3
	Below LoD	36.38	3.05	1/3	ND	ND	0/3	ND	ND	0/3
DENV-4	Above LoD	30.01	0.09	3/3	32.71	1.57	3/3	31.36	1.03	3/3
	LoD	32.58	0.47	3/3	36.55	1.01	3/3	35.00	0.47	3/3
	Below LoD	39.88	6.95	0/3	ND	ND	0/3	36.37	ND	1/3

* AVG CT = average CT value, STD = standard deviation, ND = not detected.

### Carry Over or Cross Contamination

To evaluate possible cross-contamination of samples while performing the Assay, we used a panel of DENV-1–4 at high positive (10^7^ GCE/mL) and below LoD (5×10^2^ GCE/mL) concentrations, respectively. Samples below LoD are composed of normal human serum spiked with DENV to a concentration just below the LoD. Eight replicates of the high positive and below LoD DENV concentrations were extracted using the Qiagen QIAamp DSP Viral RNA Mini Kit and tested in alternating series. All negative samples tested negative (32/32) and all DENV positive samples tested positive for DENV (32/32) (Table 5). Several below LoD samples produced CT values >37 that may correspond to detection of DENV RNA; however these results are not 100% reproducible.

**Table 5:** Assessment of the potential for carryover contamination.

	Replicate (CT value)							
	1	2	3	4	5	6	7	8
High Pos DENV-1 (10^7^ GCE/mL)	DENV-1 18.44	DENV-1 18.05	DENV-1 17.54	DENV-1 17.63	DENV-1 18.45	DENV-1 18.01	DENV-1 17.33	DENV-1 17.40
	DENV-2 ND	DENV-2 ND	DENV-2 ND	DENV-2 ND	DENV-2 38.64	DENV-2 ND	DENV-2 ND	DENV-2 ND
	DENV-3 ND	DENV-3 ND	DENV-3 ND	DENV-3 40.16	DENV-3 ND	DENV-3 ND	DENV-3 ND	DENV-3 ND
	DENV-4 ND	DENV-4 ND	DENV-4 ND	DENV-4 ND	DENV-4 ND	DENV-4 ND	DENV-4 ND	DENV-4 ND
Below LoD DENV-1 (5×10^2^ GCE/mL)	DENV-1 40.15	DENV-1 ND	DENV-1 ND	DENV-1 39.76	DENV-1 ND	DENV-1 ND	DENV-1 37.65	DENV-1 38.72
	DENV-2 ND	DENV-2 ND	DENV-2 ND	DENV-2 ND	DENV-2 ND	DENV-2 39.06	DENV-2 ND	DENV-2 ND
	DENV-3 ND	DENV-3 ND	DENV-3 ND	DENV-3 ND	DENV-3 ND	DENV-3 ND	DENV-3 ND	DENV-3 ND
	DENV-4 ND	DENV-4 ND	DENV-4 ND	DENV-4 ND	DENV-4 ND	DENV-4 ND	DENV-4 ND	DENV-4 ND
High Pos DENV-2 (10^7^ GCE/mL)	DENV-1 ND	DENV-1 ND	DENV-1 42.63	DENV-1 ND	DENV-1 ND	DENV-1 ND	DENV-1 ND	DENV-1 ND
	DENV-2 19.80	DENV-2 20.14	DENV-2 20.60	DENV-2 20.13	DENV-2 19.95	DENV-2 20.26	DENV-2 20.48	DENV-2 20.01
	DENV-3 ND	DENV-3 ND	DENV-3 ND	DENV-3 ND	DENV-3 ND	DENV-3 ND	DENV-3 ND	DENV-3 ND
	DENV-4 ND	DENV-4 ND	DENV-4 ND	DENV-4 ND	DENV-4 ND	DENV-4 ND	DENV-4 ND	DENV-4 ND
Below LoD DENV-2 (5×10^2^ GCE/mL)	DENV-1 ND	DENV-1 ND	DENV-1 ND	DENV-1 ND	DENV-1 ND	DENV-1 ND	DENV-1 40.87	DENV-1 ND
	DENV-2 ND	DENV-2 42.7	DENV-2 ND	DENV-2 37.72	DENV-2 ND	DENV-2 38.76	DENV-2 ND	DENV-2 38.54
	DENV-3 ND	DENV-3 ND	DENV-3 42.76	DENV-3 ND	DENV-3 ND	DENV-3 ND	DENV-3 ND	DENV-3 ND
	DENV-4 ND	DENV-4 ND	DENV-4 ND	DENV-4 ND	DENV-4 ND	DENV-4 ND	DENV-4 ND	DENV-4 ND
High Pos DENV-3 (10^7^ GCE/mL)	DENV-1 ND	DENV-1 42.71	DENV-1 ND	DENV-1 ND	DENV-1 ND	DENV-1 ND	DENV-1 ND	DENV-1 ND
	DENV-2 ND	DENV-2 ND	DENV-2 ND	DENV-2 ND	DENV-2 ND	DENV-2 ND	DENV-2 40.76	DENV-2 ND
	DENV-3 20.91	DENV-3 20.51	DENV-3 20.80	DENV-3 21.21	DENV-3 20.55	DENV-3 20.29	DENV-3 20.39	DENV-3 20.99
	DENV-4 ND	DENV-4 38.54	DENV-4 ND	DENV-4 ND	DENV-4 ND	DENV-4 ND	DENV-4 ND	DENV-4 ND
Below LoD DENV-3 (5×10^2^ GCE/mL)	DENV-1 ND	DENV-1 ND	DENV-1 ND	DENV-1 ND	DENV-1 ND	DENV-1 40.78	DENV-1 ND	DENV-1 ND
	DENV-2 ND	DENV-2 ND	DENV-2 ND	DENV-2 ND	DENV-2 ND	DENV-2 ND	DENV-2 ND	DENV-2 ND
	DENV-3 41.78	DENV-3 ND	DENV-3 ND	DENV-3 38.62	DENV-3 38.74	DENV-3 ND	DENV-3 ND	DENV-3 37.45
	DENV-4 ND	DENV-4 ND	DENV-4 ND	DENV-4 ND	DENV-4 ND	DENV-4 ND	DENV-4 ND	DENV-4 42.76
High Pos DENV-4 (10^7^ GCE/mL)	DENV-1 ND	DENV-1 41.68	DENV-1 ND	DENV-1 ND	DENV-1 ND	DENV-1 ND	DENV-1 ND	DENV-1 42.67
	DENV-2 ND	DENV-2 ND	DENV-2 ND	DENV-2 ND	DENV-2 38.65	DENV-2 ND	DENV-2 ND	DENV-2 ND
	DENV-3 42.70	DENV-3 ND	DENV-3 ND	DENV-3 ND	DENV-3 ND	DENV-3 ND	DENV-3 ND	DENV-3 ND
	DENV-4 17.31	DENV-4 17.13	DENV-4 17.38	DENV-4 17.26	DENV-4 17.18	DENV-4 17.15	DENV-4 17.25	DENV-4 17.07
Below LoD DENV-4 (5×10^2^ GCE/mL)	DENV-1 ND	DENV-1 ND	DENV-1 ND	DENV-1 ND	DENV-1 ND	DENV-1 ND	DENV-1 ND	DENV-1 ND
	DENV-2 ND	DENV-2 ND	DENV-2 ND	DENV-2 ND	DENV-2 ND	DENV-2 ND	DENV-2 ND	DENV-2 ND
	DENV-3 ND	DENV-3 ND	DENV-3 ND	DENV-3 ND	DENV-3 ND	DENV-3 ND	DENV-3 ND	DENV-3 ND
	DENV-4 ND	DENV-4 38.72	DENV-4 ND	DENV-4 ND	DENV-4 ND	DENV-4 ND	DENV-4 37.65	DENV-4 39.74

* ND = not detected.

### Reproducibility

Result reproducibility was evaluated at the CDC Dengue Branch and two external public health laboratories: the Puerto Rico Department of Health and the National Reference Laboratory in Costa Rica. Operator to operator, run to run, and site to site reproducibility was evaluated using five test panels which include negative, below LoD, low positive, and moderate positive samples. Cultured and quantified (GCE/mL) stocks of heat-inactivated laboratory strains DENV-1–4 were used in the panel. Two different operators tested each sample in the panel twice a day for at least 5 days. Each operator ran the entire panel twice. Every sample in the test panel was blinded for each operator, run, and device. Two different RNA extraction methods previously validated for the assay were used in the study. The CDC Dengue Branch Laboratory and one external site used the Qiagen QIAamp DSP Viral RNA Mini Kit and the second external site used the Roche MagNA Pure LC Total Nucleic Acid Isolation Kit following the manufacturer's protocol. Results of high reproducibility were obtained from each site and compared to determine the proportion agreement with the expected results (Table 6).

**Table 6:** Reproducibility summary for the CDC DENV-1–4 Real-Time RT-PCR Assay including results from three testing sites.

		AVG CT	%CV	Agreement with Expected Result	95% CI
**DENV-1**	Moderate Positive	25.52	3.41	60/60 (100%)	94.0–100
	Low Positive	31.03	2.42	58/60 (96%)	88.6–99.11
	Below LoD	38.60	3.55	13/60 (21%)	15.9–39.6
	Negative	38.54*	ND	60/60 (100%)	ND
**DENV-2**	Moderate Positive	26.32	3.91	60/60 (100%)	94.0–100
	Low Positive	31.77	3.43	57/60 (95%)	86.3–98.29
	Below LoD	38.91	3.14	14/60 (23%)	14.4–35.4
	Negative	40.32*	ND	60/60 (100%)	ND
**DENV-3**	Moderate Positive	26.01	5.96	60/60 (100%)	94.0–100
	Low Positive	31.10	4.57	60/60 (100%)	94.0–100
	Below LoD	39.25	3.59	15/60 (25%)	15.8–37.23
	Negative	39.17*	ND	60/60 (100%)	ND
**DENV-4**	Moderate Positive	25.47	4.00	60/60 (100%)	94.0–100
	Low Positive	30.62	3.89	57/60 (95%)	86.3–98.29
	Below LoD	38.62	4.17	14/60 (23%)	14.4–35.4
	Negative	ND	ND	60/60 (100%)	ND
**Neg. Control**		ND	ND	10/10 (100%)	ND

* AVG CT value is based on one or two samples.

% CV = coefficient of variation.

### Prospective Study

86 acute phase (0–5 days of illness: 8 DPO = 1; 12 DPO = 2; 22 DPO = 3; 29 DPO = 4; 15 DPO = 5; Avg DPO = 3.3, Std Dv = 1.2) serum samples were tested in the multiplex format of the assay and corroborated by E gene sequencing. A total of 48 samples produced positive sequence results, of which 47 were positive on the RT-PCR Assay (16 DENV-1, 11 DENV-2, 5 DENV-3 and 15 DENV-4). The single discordant sample was identified as DENV-3 by sequencing. The 38 samples with RT-PCR negative results in the Assay were also negative by sequencing. Applying sequencing as the reference method, the RT-PCR Assay had a 97.92% positive agreement and 100% negative agreement (Table 7). All samples tested positive for the RP control.

**Table 7:** Agreement between CDC DENV-1–4 Real Time RT-PCR and sequencing in samples from suspected dengue patients.

Multiplex CDC DENV-1–4 Real-Time RT-PCR Assay Comparison Results				
		Reference Method (Sequencing)		
		Positive	Negative	Total
**CDC DENV-1–4 Real-Time RT-PCR Assay**	Positive	47	0	47
	Negative	1*	38	39
	Total	48	38	86

	Value	95% Confidence Interval
**Positive percent agreement**	97.92%	89.10–99.63
**Negative percent agreement**	100%	90.82–100

* One sample was negative on the CDC-DENV-1–4 RT-PCR assay which was positive for DENV-3 sequencing. All other negative RT-PCR samples were also negative by E gene sequencing.

### Retrospective Studies

Of the 371 serum samples (0–5 days of illness: 36 DPO = 1; 56 DPO = 2; 81 DPO = 3; 115 DPO = 4; 83 DPO = 5; Avg DPO = 3.4, Std Dv = 1.2) tested in this evaluation, 102 had IgM anti-DENV seroconversion in the convalescent specimen. 100 out of these 102 samples were positive in the RT-PCR Assay using the multiplex format (98.04% positive agreement). Of the 2 discordant samples, one was positive for DENV-3 by singleplex and further confirmed by E gene sequencing. The other discordant specimen was repetitively negative by multiplex and singleplex, but DENV-3 positive by sequencing. Of the 269 samples with an IgM anti-DENV negative convalescent sample, 265 were negative by the RT-PCR Assay (98.51% negative agreement; Table 8); however, the remaining four samples were positive by sequencing (2 DENV-3 and 2 DENV-4). All serotypes were identified in this study (26 DENV-4, 29 DENV-2, 22 DENV-3 and 23 DENV-4) and all 371 samples had a RP positive control reaction.

**Table 8:** Agreement between CDC DENV-1–4 Real Time RT-PCR and seroconversion in samples from suspected dengue patients.

Multiplex CDC DENV-1–4 Real-Time RT-PCR Assay Comparison Results				
		Reference Method (IgM Conversion)†		
		Positive	Negative	Total
**CDC DENV-1–4**	Positive	100*	4***	104
**Real-Time RT-PCR Assay**	Negative	2**	265	267
	Total	102	269	371

	Value	95% Confidence Interval
**Positive percent agreement**	98.04%	93.13–99.46
**Negative percent agreement**	98.51%	96.24–99.42

* One DENV-1 case was positive on the CDC DENV-1–4 Real-Time RT-PCR Assay (multiplex) and confirmed positive by IgM conversion, but not sequenced effectively enough to produce an interpretable result.

** Two samples were negative by the CDC-DENV-1–4 Real-Time RT-PCR Assay (multiplex). One of these samples was DENV-3 positive by the CDC-DENV-1–4 Real-Time RT-PCR Assay (singleplex) and was further confirmed DENV-3 positive by sequencing. The other sample was confirmed DENV-3 positive by sequencing.

*** Four samples were positive RT-PCR results and did not confirmed by seroconversion. Two of these samples obtained positive results for DENV-3 and the other two samples were DENV-4 positive and these results were confirmed by E gene sequencing.

† All instances of IgM conversion were demonstrated in paired acute and convalescent samples.

### Performance in Fresh vs. Frozen Specimens

To determine the effect of temperature variations on Assay performance, we compared detection of DENV-spiked human serum samples after multiple freeze/thaw cycles. Moderate and low positive dilutions were prepared in triplicate, frozen at −80°C for 24 hours, and subjected to five consecutive freeze/thaw cycles. Once thawed, every sample was processed according to the Assay protocol. We obtained a 100% qualitative agreement between the initial and post freeze/thaw cycle (1, 2, 3, 4, and 5) detection results (Supporting Figure S1).

## Discussion

Early diagnosis of dengue based on a single serum sample obtained in the febrile phase of the illness has been a challenge for the public health and clinical communities. The commonly used IgM anti-DENV test is only capable of diagnosing dengue upon the rise of the antibody response, usually after 4–5 days upon illness onset. NS1 antigen detection tests are not serotype-specific and recent evaluations suggest that they are not highly sensitive and are best utilized at defervescence [30], [49]. The presence of DENV viremia during the first 5 days of illness renders molecular methods optimal for dengue diagnostics. The development of the CDC DENV-1–4 Real-Time RT-PCR Assay for the detection and serotyping of DENV facilitates dengue diagnosis in both the clinical and public health surveillance settings. In addition, the evaluation and approval of this diagnostic platform by the FDA provides a benchmark for other DENV detection assays.

The CDC DENV-1–4 Real-Time RT-PCR Assay was developed over the same diagnostic platform used by other CDC-developed, FDA-approved diagnostic platforms including the CDC Human Influenza Virus Real-time RT-PCR Diagnostic Panel (cat# KT0096). During and after the influenza A H1N1 pandemic in 2009, many national and international laboratories acquired the CDC flu diagnostic platform through a dedicated network for supplies distribution, including the recommended ABI 7500Dx thermocycler. The CDC DENV-1–4 Real Time RT-PCR Assay was designed to be compatible and utilize the same reagents and equipment, such that laboratories that previously acquired the CDC flu platform can now acquire the DENV assay with minimal investment through a similar network for supplies. An important caveat is that the dengue platform requires a 4-color real time thermocycler even if the assay is run in singleplex format. Since the approval of the CDC DENV-1–4 RT-PCR assay in May 2012, more than 100 public health laboratories worldwide have begun to run this Assay, resulting in decentralization and uniformity of dengue diagnosis. The implementation of this sensitive test for early diagnosis of dengue strengthens laboratory surveillance and allows the diagnosis and management of a higher proportion of dengue cases faster, possibly defining outbreaks more promptly.

Our evaluation shows that the CDC DENV-1–4 Real Time RT-PCR Assay confirms more than 90% of dengue cases. Although the analytical sensitivity of this Assay is comparable among other real time RT-PCR-based diagnostic assays, this Assay was designed to detect viruses from a wide geographical distribution identified during recent outbreaks, significantly enhancing the capacity to detect a wide range of DENV genetic variants. This design distinguishes the CDC Assay from other published assays that are mostly based on laboratory-adapted DENV strains originally isolated decades ago and belonging to extinct or rarely transmitted genotypes [34], [36], [38], [50]. The ability to detect a wide range of international DENV strains provides diagnostic laboratories the capacity to detect DENV introductions or resolve traveler-associated cases.

Our sequence analysis considered the 5–6% genomic difference between genotypes and predicted that the significant number of mismatches would impair the successful detection of many contemporary strains when an assay is designed based on prototype strains. In order to achieve detection of DENV strains with high genetic divergence, we compared the target genetic sequence of a large number of contemporary DENV strains from a wide geographic distribution to the primers and probes of the CDC DENV-1–4 Real-Time RT-PCR Assay. Oligonucleotide positions with frequent mismatches were substituted with degenerate nucleotides; therefore the primer and probe mix contains a cocktail of oligonucleotides with 2 or more different nucleotide options at the degenerate base position. This cocktail of oligonucleotide sequences within the same primer mix is expected to increase the probability of finding a complete match between primer and target virus sequence, consequently increasing the likelihood of detecting virus strains with a high degree of genetic divergence. Other assays have incorporated the use of nucleic acid degeneracy, however these assays were based on laboratory-adapted strains [51], required long, labor-intensive protocols [52], [53] or are not capable of serotype identification [54].

These evaluations also demonstrated the high specificity of this Assay which did not produce false DENV positive or false negative results even in samples from patients affected by diseases in the differential diagnosis of dengue nor the presence of interfering substances at pathogenic levels. The analytical performance of this Assay showed comparable sensitivity detecting the four DENV serotypes in both the singleplex and multiplex formats, which allows users to run only one test per sample and maximize use of reagent, consumables and equipment. Minimal cross-reactivity between oligonucleotides in the multiplex reaction is expected in this type of format and was observed at high CTs (>37) in some non-template controls. This could also explain the differences in sensitivity (≤1 log) between serotypes and formats. The PCR reaction in this Assay targets a different genomic region for each serotype; therefore different reaction efficiencies, which may affect amplification and sensitivity, are expected. Users of this Assay will have the capability of diagnosing dengue with confidence among undifferentiated or acute febrile patients. Moreover, we show that the capacity of this Assay to detect DENV RNA even on samples that have endured multiple freeze-thaw cycles, thus demonstrating that this Assay can be used with confidence on archival or mishandled samples. Two independent indicators were used to assess the performance of the RT-PCR Assay: sequencing of the E gene from DENV RNA in the same sample where the RT-PCR was performed, and IgM anti-DENV seroconversion in paired samples from each case. Of the 4 non-concordant, a possible explanation is that IgM anti-DENV was not detected in the convalescent samples due to low levels of IgM antibodies in secondary DENV infections. Further studies are needed to determine the true frequency of this observation and establish the proportion of RT-PCR positive cases beyond the first 5 days of illness. Importantly, the sensitivity of the E gene sequencing has not been extensively evaluated for its potential as a diagnostic test or its sensitivity and it has served in this study as an additional comparator to confirm DENV serotype detection in acute samples due to lack of more sensitive tests for dengue.

The inclusion of the RP control in the RT-PCR Assay further facilitates results interpretation. The RP reaction detects human RNA and could potentially detect DNA; however the viral RNA extraction eliminates must of the contaminating DNA. Importantly, samples with a positive DENV result should be considered positive even when they may occasionally have a RP negative control. However a DENV negative result on a sample with RP-negative should be considered inconclusive since this result is likely due to poor RNA extraction or sample quality; such samples should be retested. Despite the high sensitivity, a negative RT-PCR result does not preclude the diagnosis of dengue and should not be used as a sole basis for treatment or other patient management decisions. If results of the DENV RT-PCR Assay are negative during an acute dengue-like illness, anti-DENV IgM testing should be considered.

The adaptation of the CDC DENV-1–4 Real-Time RT-PCR Assay for use on the ABI 7500 FAST Dx thermocycler using readily available reagents provides the end-user a dengue diagnostic capability over an operating platform already available in many clinical and reference diagnostic laboratories. Development of diagnostic platforms evaluated by a rigorous regulatory authority such as the FDA should encourage and benchmark development of other assays to meet the needs of dengue endemic areas. It is clear that platforms with similar performance characteristics are needed for different clinical care setting in order to improve case management and disease surveillance. Laboratories equipped with other thermocyclers are encouraged to validate this Assay within their particular technical capabilities. For a high incidence disease such as dengue, which could potentially become a vaccine-preventable disease in the future, diagnostic tests with similar performance to the CDC RT-PCR Assay are needed for resource-limited areas that are instrument independent or that require minimal instrumentation.

## Supporting Information

Checklist S1 Checklist prepared to indicate the specific page in which each part of the study can be found in this manuscript.
(DOCX)

Figure S1 Assay performance in fresh vs. frozen samples. Moderate (black circle) and low (black square) positive concentrations of laboratory-adapted DENV strains diluted in serum were frozen at −80°C for 24 hrs and subject to five consecutive freeze/thaw cycles. Detection measurements are shown in CT values and error bars indicate standard deviation. Dashed line shows the threshold of positivity at CT = 37.
(TIF)

Flowchart S1 Two flowcharts were created to indicate the diagnostic algorithm used in the Retrospective and Prospective studies in which the performance of the CDC DENV-1–4 Real Time RT-PCR Assay was evaluated.
(PDF)

Table S1 DENV E gene amplification and sequencing primers. List of all primer sequences used for E gene amplification and sequencing. Corresponding serotype, specific primer function and final concentration per reaction are indicated. Every primer is labeled with serotype and genome position. Reverse primers are labeled (cD).
(DOCX)

Table S2 Performance of the CDC DENV-1–4 Real Time RT-PCR Assay using different viral RNA extraction methods. Viral RNA from serial dilutions of stock quantitated DENV-1–4 was extracted using several viral RNA extraction kits. The lowest virus titer detected is indicated in genome copy equivalents per mL (GCE/mL).
(DOCX)
