# Correction: Analytical and Clinical Performance of the CDC Real Time RT-PCR Assay for Detection and Typing of Dengue Virus

**DOI:** 10.1371/annotation/ae27d48b-025f-47ce-8427-4af59f821ad7

**Published:** 2013-07-31

**Authors:** Gilberto A. Santiago, Edgardo Vergne, Yashira Quiles, Joan Cosme, Jesus Vazquez, Juan F. Medina, Freddy Medina, Candimar Colón, Harold Margolis, Jorge L. Muñoz-Jordán

The image and legend for Figure 1 is incorrect. The correct image can be found at the following URL:

**Figure:**
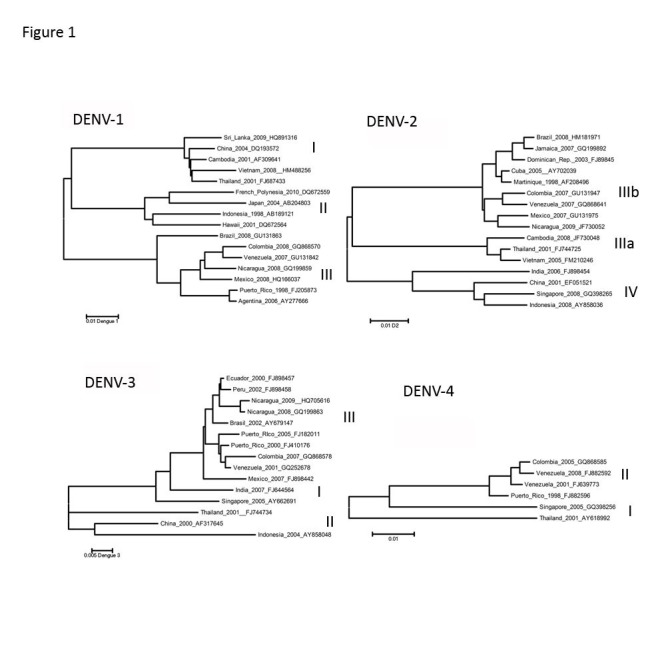


The correct legend is as follows:

Figure 1. Maximum likelihood phylogeny of contemporary DENV strains

Representative DENV strains from currently circulating genotypes considered for primer and probe modifications. Full genome sequences were used. Each taxa is labeled: country of origin, year of isolation, and GenBank accession number. Due to figure space limitations, only a representative subset of strains were used to generate these ML trees. DENV-1 Asian (I), South Pacific (II), and American-African (III) genotypes [55], DENV-2 Asian I (IIIa), American-Asian (IIIb), and Cosmopolitan (IV) genotypes [42], DENV-3 South Pacific (I), Thailand (II), and Indian Subcontinent (III) genotypes [55,56], DENV-4 Southeast (I) and Indonesian (II) genotypes [56]. 

