# Supplementary figures and images for: Analytical and Clinical Performance of the CDC Real Time RT-PCR Assay for Detection and Typing of Dengue Virus
Source: PLoS Negl Trop Dis. 2013 Jul 11;7(7):e2311. doi: 10.1371/journal.pntd.0002311 (PMC3708876; doi:10.1371/journal.pntd.0002311)

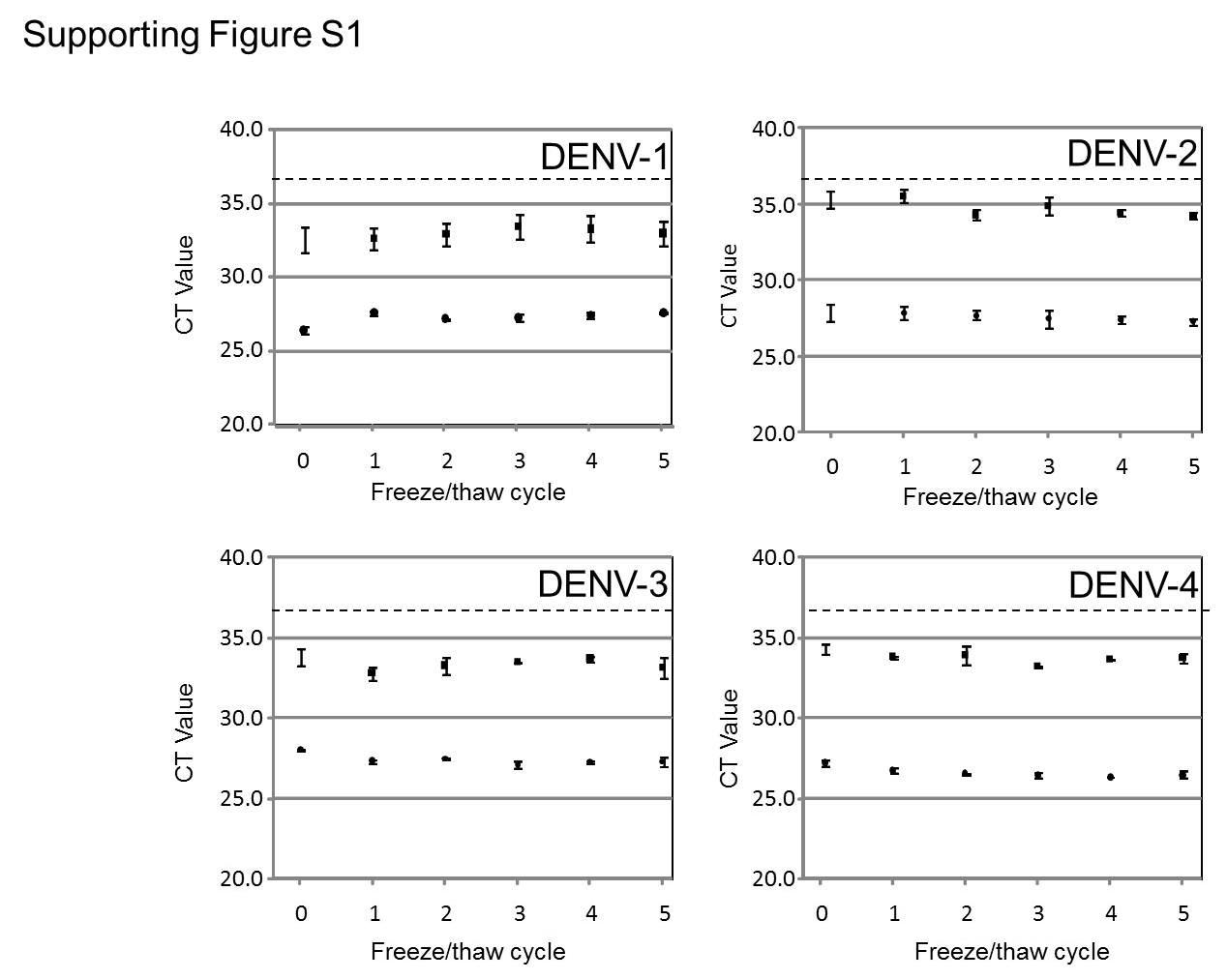

Supplement: Figure S1 — Assay performance in fresh vs. frozen samples. Moderate (black circle) and low (black square) positive concentrations of laboratory-adapted DENV strains diluted in serum were frozen at −80°C for 24 hrs and subject to five consecutive freeze/thaw cycles. Detection measurements are shown in CT values and error bars indicate standard deviation. Dashed line shows the threshold of positivity at CT = 37. (TIF) [file pntd.0002311.s002.tif]

# Retrospective Study

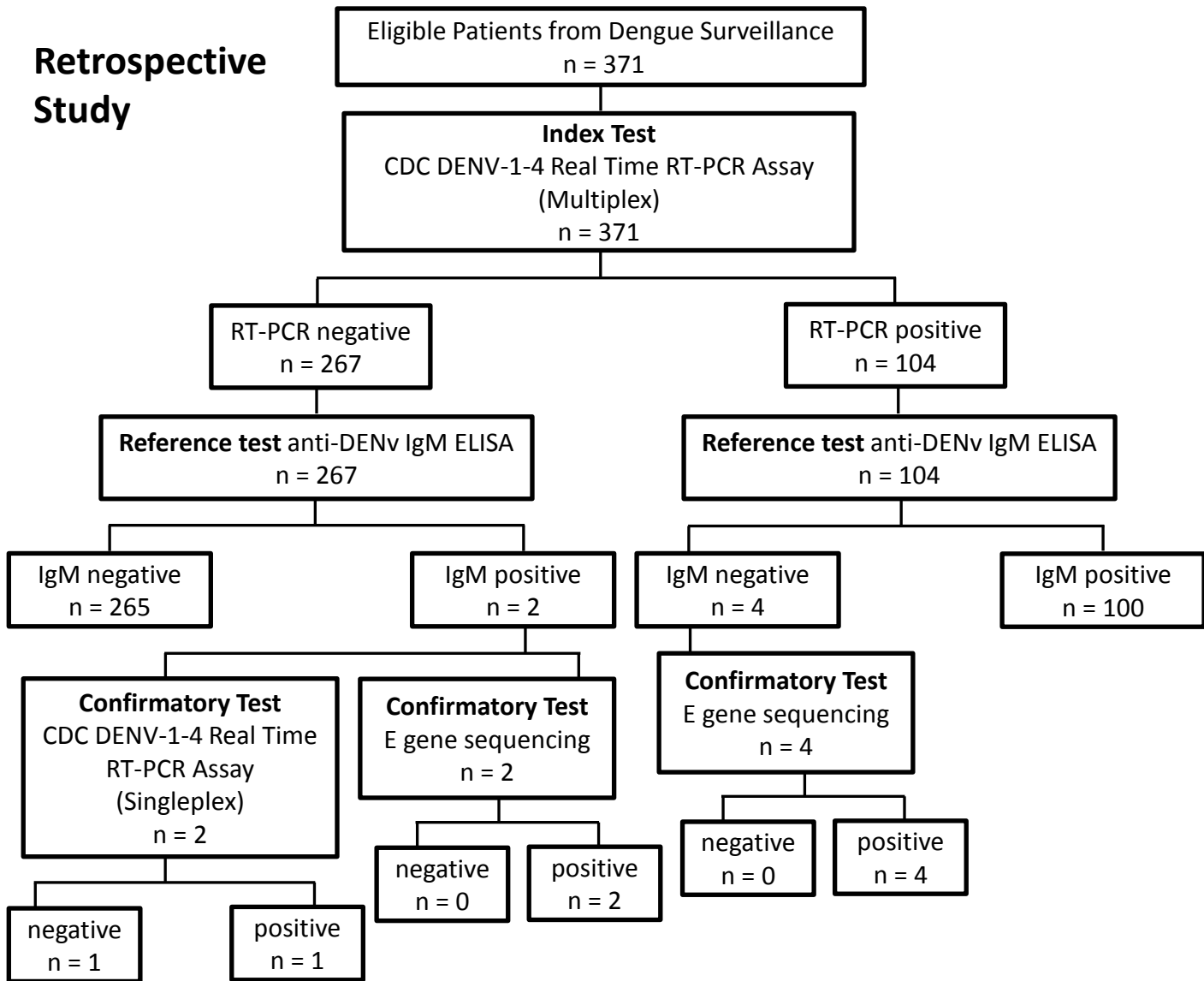

# Prospective Study

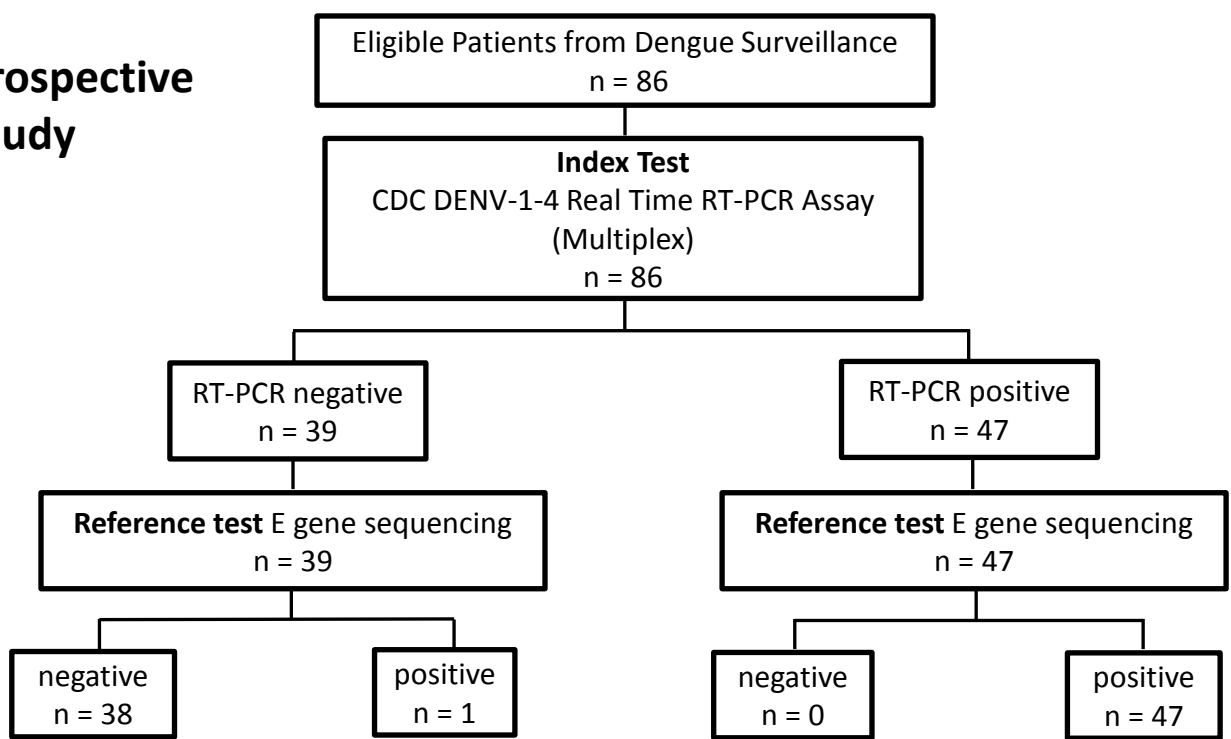

Supplement: Flowchart S1 — Two flowcharts were created to indicate the diagnostic algorithm used in the Retrospective and Prospective studies in which the performance of the CDC DENV-1–4 Real Time RT-PCR Assay was evaluated. (PDF) [file pntd.0002311.s003.pdf]
