# Supplementary material for: Analytical and Clinical Performance of the CDC Real Time RT-PCR Assay for Detection and Typing of Dengue Virus
Source: PLoS Negl Trop Dis. 2013 Jul 11;7(7):e2311. doi: 10.1371/journal.pntd.0002311 (PMC3708876; doi:10.1371/journal.pntd.0002311)
Supplement: Table S1 — DENV E gene amplification and sequencing primers. List of all primer sequences used for E gene amplification and sequencing. Corresponding serotype, specific primer function and final concentration per reaction are indicated. Every primer is labeled with serotype and genome position. Reverse primers are labeled (cD). (DOCX) [file pntd.0002311.s004.docx]

**Supporting Table S1:** DENV E gene amplification and sequencing primers.

| **Serotype** | **Name** | **Sequence** | **Function** | **Final**  **Concentration** |
| --- | --- | --- | --- | --- |
| 1 | D1-1 | AGTTGTTAGTCTACGTGGACCGAC | amplification | 400nM |
| 1 | cD1-2086 | CACCTGCTCCTACCACGATGTAGC | amplification | 400nM |
| 1 | D1-1880 | CAGCATGGAACTGTTCTGGTGCAG | amplification | 400nM |
| 1 | cD1-4196 | GAGGAGTGAACTTAGTAGGATGCT | amplification | 400nM |
| 1 | cD1-973 | CCACGAAGTCTCTGTTGCCTATT | sequencing | 165nM |
| 1 | D1-890 | AAGGGATCATTTTCATTTTG | sequencing | 165nM |
| 1 | D1-1003 | GTGGTACTGGAGCATGGAAGTTGC | sequencing | 165nM |
| 1 | cD1-1446 | CGTAGGAGCTTGAGGTGTTATGGT | sequencing | 165nM |
| 1 | D1-1395 | TGAGACTACAGAACATGGAACAAC | sequencing | 165nM |
| 1 | cD1-1923 | TTCATATTTAACCTGCACCAGAAC | sequencing | 165nM |
| 1 | D1-2141 | GAAGCAACTGCCCGAGGAGCACGA | sequencing | 165nM |
| 2 | D2-841 | ATGATGGCAGCAATCCTGGCATAC | amplification | 400nM |
| 2 | cD2-2670 | CTCCTGTCATAATAGTTAACTTCACC | amplification | 400nM |
| 2 | cD2-771 | ATGTTTCCAGGCCCCTTCTGATGAC | sequencing | 165nM |
| 2 | cD2-1387 | CATGTTTTCCTGTGTCATTTCCGAC | sequencing | 165nM |
| 2 | D2-1416 | GGAAATCAAAATAACACCACAGAGTTCC | sequencing | 165nM |
| 2 | cD2-2074 | TGATGATGTAGCTGTCTCCGAATG | sequencing | 165nM |
| 2 | D2-2207 | TCCTTGGGAGGAGTGTTTACATCT | sequencing | 165nM |
| 3 | D3-27 | AGTTGTTAGTCTACGTGGACCGACAAG | amplification | 400nM |
| 3 | cD3-1345 | GTGTGCACTGTGATGATGACGGTG | amplification | 400nM |
| 3 | D3-1325 | GAATCAATAGAGGGAAAAGTGGTGC | amplification | 400nM |
| 3 | cD3-2678 | GACCCCAGTTATGTCGCCTACAAC | amplification | 400nM |
| 3 | D3-933 | TTAATGCTGGTTACCCCATCCATGAC | sequencing | 165nM |
| 3 | cD3-947 | CTTCCACAAAATCTCTGTTTCCTACTCC | sequencing | 165nM |
| 3 | cD3-1336 | CTGTGATGATGACGGTGTATTTGAGG | sequencing | 165nM |
| 3 | D3-1331 | CAATAGAGGGAAAAGTGGTGCAACATG | sequencing | 165nM |
| 3 | D3-1798 | GCACTTAAAATGTAGACTCAAGATGGAC | sequencing | 165nM |
| 3 | cD3-1819 | GCTACTCAAGCACATTGCATAGCTC | sequencing | 165nM |
| 3 | D3-2240 | GAATTCATTAGGGAAAATGGTCCACC | sequencing | 165nM |
| 3 | cD3-2253 | CCACCAAATAGGGCTGTGTAAGCAC | sequencing | 165nM |
| 4 | D4-1 | AGTTGTTAGTCTGTGTGGACCGAC | amplification | 400nM |
| 4 | cD4-2520 | CTGTTCTGTCCAAGTGTGC | amplification | 400nM |
| 4 | D4-836 | TCGCGCTCTTGGCAGGATTTATGG | sequencing | 165nM |
| 4 | cD4-1010 | AGATCGACCCATGCTCCACCTG | sequencing | 165nM |
| 4 | D4-1237 | TGGGGCAATGGCTGTGGCTTGTTT | sequencing | 165nM |
| 4 | D4-1424 | CAGCCACGATAACTCCCAGGTCAC | sequencing | 165nM |
| 4 | cD4-1490 | AGTGTTAGTTCTCCATAGTCC | sequencing | 165nM |
| 4 | D4-1780 | GCAGGACATCTCAAGTGCAAAGTC | sequencing | 165nM |
