# Supplementary material for: Analytical and Clinical Performance of the CDC Real Time RT-PCR Assay for Detection and Typing of Dengue Virus
Source: PLoS Negl Trop Dis. 2013 Jul 11;7(7):e2311. doi: 10.1371/journal.pntd.0002311 (PMC3708876; doi:10.1371/journal.pntd.0002311)
Supplement: Table S2 — Performance of the CDC DENV-1–4 Real Time RT-PCR Assay using different viral RNA extraction methods. Viral RNA from serial dilutions of stock quantitated DENV-1–4 was extracted using several viral RNA extraction kits. The lowest virus titer detected is indicated in genome copy equivalents per mL (GCE/mL). (DOCX) [file pntd.0002311.s005.docx]

**Supporting Table S2:** Performance of the CDC DENV-1-4 Real Time RT-PCR assay using different viral RNA extraction methods.

| **RNA Extraction Method** | **Automation** | **Lowest Virus Titer of Detection** | | | |
| --- | --- | --- | --- | --- | --- |
|  |  | **DENV-1** | **DENV-2** | **DENV-3** | **DENV-4** |
| Qiagen QIAamp DSP Viral RNA Mini Kit and (61904 and 9001292) | Manual or in Qiagen QIAcube Instrument (9001292) | 3.1 x 10^3^ | 3.2x10^3^ | 3.2X10^3^ | 8.9x10^2^ |
| Qiagen QIAamp Viral RNA Kit (52904 and 52906) | Manual or in Qiagen QIAcube Instrument (9001292) | 3.2 x 10^3^ | 9.8x10^2^ | 3.3X10^3^ | 3.1x10^3^ |
| Roche MagNA Pure LC total Nucleic Acid Isolation Kit (03 038 505 001) | MagNA Pure LC 2.0 instrument (05 197 686 001) | 2.6 x 10^3^ | 1.2x10^3^ | 2.2X10^3^ | 2.3x10^3^ |
| QIAamp Virus BioRobot MDx Kit (965652) | BioRobot Universal System (9001094) | 2.5 x 10^3^ | 2.4x10^3^ | 8.1X10^2^ | 1.1x10^3^ |
| MagAttract Virus Mini M48 Kit (955336) | BioRobot M48 System (9000708) | 9.1 x 10^3^ | 2.8x10^3^ | 1.9X10^3^ | 2.3x10^3^ |

*titers in GCE/mL
